# Antioxidant and Hepatoprotective Properties of Tofu (*Curdle Soymilk*) against Acetaminophen-Induced Liver Damage in Rats

**DOI:** 10.1155/2013/230142

**Published:** 2013-02-28

**Authors:** Ndatsu Yakubu, Ganiyu Oboh, Amuzat Aliyu Olalekan

**Affiliations:** ^1^Department of Biochemistry, Ibrahim Badamasi Babangida University, Niger State, Lapai, Nigeria; ^2^Department of Biochemistry, Federal University of Technology, Ondo State, Akure, Nigeria

## Abstract

The antioxidant and hepatoprotective properties of tofu using acetaminophen to induce liver damage in albino rats were evaluated. Tofus were prepared using calcium chloride, alum, and steep water as coagulants. The polyphenols of tofu were extracted and their antioxidant properties were determined. The weight gain and feed intake of the rats were measured. The analysis of serum alanine aminotransferase (ALT), alkaline phosphatase (ALP), aspartate aminotransferase (AST), and lactate dehydrogenase (LDH) activities and the concentrations of albumin, total protein, cholesterol, and bilirubin were analyzed. The result reveals that the antioxidant property of both soluble and bound polyphenolic extracts was significantly higher in all tofus, but the steep water coagulated tofu was recorded higher. Rats fed with various tofus and acetaminophen had their serum ALP, ALT, AST, and LDH activities; total cholesterol; and bilirubin levels significantly (*P* < 0.05) reduced, and total protein and albumin concentrations increased when compared with basal diet and acetaminophen administered group. Therefore, all tofus curdled with various coagulants could be used to prevent liver damage caused by oxidative stress.

## 1. Introduction

Reactive oxygen species (ROS) have been implicated in more than 100 diseases [1]. Foods (tubers, grains, fruits, and vegetables) provide a wide variety of ROS-scavenging antioxidants such as phytochemical and antioxidant vitamins [2, 3]. The increased consumption of fruits and vegetables, containing high levels of phytochemicals, has been recommended to prevent or reduce oxidative stress in the human body [2–4]. The natural antioxidant defense mechanisms can be insufficient and hence dietary intake of antioxidant components is important and recommended [5]. 

 Liver disease is a worldwide problem. Conventional drugs used in the treatment of liver diseases are sometimes inadequate and can have serious adverse effects [6]. Soybeans are inexpensive and serve as high quality protein source. Soymilk and tofu consumption is increasing in Nigeria due to animal diseases such as mad cow disease, global shortage of animal protein, strong demand for healthy (cholesterol-free and low in saturated fat) and religious halal food, and economic reasons [7]. The greater acceptance of soy foods by the general population is due to increased recognition of the health benefits of soy foods, especially by those who want to reduce their consumption of animal products [8]. Tofu, also known as soybean curd, is a soft cheese-like food made by curdling fresh hot soymilk with a coagulant [9]. Traditionally, in Nigeria, it is produced by curdling fresh hot soymilk with CaCl_2_, MgSO_4_ alum, or steep water (effluence from pap produced from maize) [10–12]. Tofu is rich in proteins; low in saturated fats; higher in polyunsaturated fatty acids; cholesterol-free; and a good source of *β*-vitamin, minerals, isoflavones, and antioxidants (carotenoids, vitamins C and E, phenolic and thiol (SH) compounds, and essential amino acids) [8, 12]. It has been demonstrated that phenolic compounds are effective antioxidants, due to the formation of stable phenoxyl radical [13], and that flavonoids have potent antioxidant activities by scavenging hydroxyl radicals, superoxide anions, and lipid peroxy radicals [14]. Soybean products reduce the risk of heart diseases by lowering levels of oxidized cholesterol, which is taken up more rapidly by coronary artery walls to form dangerous plaques [15]. Previous research has shown that soy consumption reduces cholesterol in general while also decreasing the amount of bad cholesterol (low-density lipoprotein, LDL) in the body and maintaining the amount of good cholesterol (LDL) [16, 17]. 

 Acetaminophen is a commonly used and safe analgesic drug, which is known to cause centribular necrosis upon overdose [18]. Its toxicity accounts for many emergency hospital admission and continues to be associated with high mortality. The hepatotoxic effect of acute paracetamol overdose is well known and has been extensively reviewed [19]. However, tofu (soybean product) contains compounds that are valuable antioxidants and protecting molecules, which can trap or destroy free radicals and subsequently protect us from damage due to oxidative stress [9]. In view of this, people have started to take an interest in tofu consumption due to its good nutritional and health benefit to human. Therefore, this research is designed to evaluate its polyphenol distribution and the antioxidant properties in tofu produced using different coagulants, and to compare its hepatoprotective properties on acetaminophen-induced toxicity in rat's liver.

## 2. Materials and Methods

The soybeans (*Glycine max*), TGX923-1E variety, were obtained directly from Ibrahim Badamasi Babangida University, Lapai experimental farm in Niger State, Nigeria. The alum and calcium salt (CaCl_2_) were industrial grade, while the steep water was collected from domestically processed pap. The water used in the analysis was glass distilled. The weaning albino rats used were of the same litter origin obtained from the rat colony of the Department of Biochemistry, University of Ilorin, Nigeria.

### 2.1. Tofu Preparation

Soybeans (2.0 kg) were soaked in water (6 litres) at 20–40°C for 9 hours. The soaked beans were drained, weighed, and ground with grinder; tap water was added at a ratio of 6 : 1 with raw bean and then filtered to separate soy cake from soymilk. The soymilk was subsequently heated to 98°C and maintained for 1 minute before delivering to the mix tank. When cooled to 87°C, 1 litre of soymilk was mixed at 420 rpm with each of the coagulants (50 mL). The mixed solutions were held for 5 seconds and then filled onto tofu trays and allowed to coagulate for 10 minutes. The bean curd was pressed after which the tofu weight was recorded. Tofu was stored in water at 4°C overnight prior to analysis.

### 2.2. Animals

Twenty-five of 3-week old strain albino rats with an average weight of 50.2 g were used in this study. They were obtained from the animal house of the College of Medicine, University of Ilorin, Kwara State, Nigeria. The animals were housed in metabolic cage in the laboratory under ambient temperature and 12-hour light and dark periodicities. They were fed with commercial rat pellets (Neimeth Livestock Feeds Ltd., Ikeja) and water *ad libitum* and allowed to acclimatize for 2 weeks. Animal experiments were conducted in accordance with the internationally accepted principle for laboratory animal use and care [20].

### 2.3. Extraction of Free Soluble Polyphenols

Free soluble phenols were extracted using the method modified by Nwanna and Oboh [21]. About 200 g of each tofu was homogenized in 80% acetone (1 : 2 w/v) separately using chilled Waring blender for 5 minutes. Thereafter, the homogenates were filtered through Whatman number 2 filter paper on a Buchner funnel under vacuum. The residues were kept for extractions of bound phenols. The filtrates were evaporated using a rotary evaporator under vacuum at 45°C until 90% of the filtrates had been evaporated. The extracts were frozen at −40°C. 

### 2.4. Extraction of Bound Polyphenols

These were extracted using the method modified by Nwanna and Oboh [21]. The bound phenolic contents were extracted from the residue from the free soluble polyphenol extracts. The drained residues from soluble free extraction were hydrolyzed directly with 20 mL of 4 M NaOH at room temperature for 1 hour with shaking. The mixture was acidified to PH 2 with conc. HCl and extracted six times with ethyl acetate. The ethyl acetate fractions were evaporated at 45°C under vacuum to dryness.

### 2.5. Antioxidant Activity

#### 2.5.1. Total Phenol Content

These were determined by the modified method of Nwanna and Oboh [21]. About 0.5 mL of each extract was dissolved in 20 mL of 70% acetone with equal volume of water; 0.5 mL Folin-Cioalteus reagent and 2.5 mL of sodium carbonate were subsequently added and the absorbance was measured after 40 minutes at 725 nm, using tannic acid as standard. 

#### 2.5.2. Reducing Property

These were determined by assessing the ability of the tofu extracts to reduce FeCl_3_ solution using modified method of Nwanna and Oboh [21]. About 2.5 mL of each extract was dissolved in 20 mL of methanol and mixed with 2.5 mL of 200 M sodium phosphate buffer (PH 6.6) and 2.5 mL of 100% potassium ferricyanide. The mixtures were incubated at 50°C for 20 minutes; thereafter 2.5 mL, 10%, trichloroacetic acid was added and subsequently centrifuged at 650 rpm for 10 minutes; 5 mL of the supernatant was mixed with equal volume of water and 1 mL of 0.1% ferric chloride. The absorbances were then measured at 700 nm, and a higher absorbance indicates a higher reducing power. 

#### 2.5.3. Free Radical Scavenging Ability

The free radical scavenging ability of the soluble free and bound extracts against DPPH (1, 1-diphenyl-2-picrylhydrazyl) free radical was also evaluated [22]. About 1 mL of the extract was dissolved in 20 mL methanol and mixed with 1 mL of 0.4 M methanolic solution containing 1, 1-diphenyl-2-picrylhydrazyl (DPPH) radicals. The mixtures were left in the dark for 30 minutes before measuring the absorbance at 516 nm.

### 2.6. Experimental Design

Animals were weighed and randomly assigned into five groups, namely,

 normal control group (*n* = 5) was placed on a basal diet of 20 g/day only;
 negative control group (*n* = 5) was placed on 20 g/day and 100 mg/100 g/day of basal diet and acetaminophen orally administered, respectively;
 treated group 1 (*n* = 5) was placed on steep water tofu (20 g) and acetaminophen (100 mg/100 g/day) orally administered, respectively;
 treated group 2 (*n* = 5) was placed on alum tofu (20 g) and acetaminophen (100 mg/100 g/day) orally administered, respectively;
 treated group 3 (*n* = 5) was placed on calcium tofu (20 g/day) and acetaminophen (100 mg/100 g/day) orally administered, respectively.

The experimental period was 14 days. During the experiment, the weights of the rats were measured two times in a week during the 2 weeks of the experiment.

### 2.7. Measurements

The mean average weight of the rats was determined at the beginning of the experiment at every 2 days. The weight of the rats was determined using weighing scale (OHAUS MODEL Cs 5000, CAPACITY 500 × 2 g). This was done by placing a container on the scale, with the balance adjusted to zero, after which the rats in each group were placed into container and the measurement taken [23]. Total feed consumed in percentage was calculated using the following formula:

total feed consumed (%) = 100 (feed supplied (20 g) − leftover (g)/feed supplied (20 g). 

total weight gain (TWG) in percentage was calculated as

 total weight gain (%) = 100 × (final weight − initial weight)/initial weight [24]. 

On the 15th day, the rats were killed by cervical dislocation, the bloods collected were centrifuged, and the supernatants were collected for the assessment of liver function. In addition, the livers were harvested for histopathological analysis.

#### 2.7.1. Assessment of Liver Function

The serum was used for the assay of marker enzymes (aspartate aminotransferase (AST), alanine aminotransferase (ALT), alkaline phosphatase (ALP), lactate dehydrogenase (LDH), and bilirubin) concentrations using Roche Modular Autoanalyzer. Total protein, albumin, sugar, and total cholesterol were analyzed by using standard randox laboratory kits.

### 2.8. Statistical Analysis

The results were reported as mean ± SEM (standard error of mean). One-way analysis of variance and least significant difference (LSD) test were used to evaluate the significant difference. Probability levels of less than 0.05 were considered significant.

## 3. Results 

Table 1 shows the result of antioxidants properties of the tofu studied. The results revealed that the steep water coagulated tofu significantly recorded high total phenols (15.0 ± 2.24%), followed by the alum-coagulated tofu (13.0 ± 2.08), and the calcium salt coagulated tofu appeared significantly higher in free radical scavenging activities of the free soluble phenol (65.5 ± 4.67%). The alum coagulated tofu (47.3 ± 3.97%) and the steep water had the least (38.2 ± 3.57%). Conversely, steep water coagulated tofu significantly recorded higher free radical scavenging activity of the bond phenol (69.1 ± 4.80%), followed by alum coagulated tofu (56.5 ± 3.36%), and calcium salt coagulated tofu had the lowest (27.3 ± 3.02%) free radical scavenging activity of the bound phenol. Also, the reducing power of free soluble and bound phenol of steep water and calcium salt coagulated tofu were not significantly different (0.4 ± 0.20), while alum coagulated tofu had the reducing power of free soluble phenol (0.3 ± 0.17) to be lower than that of bound phenol (0.5 ± 0.30).

 The result of total feed intake and total weight gain are presented in Table 2 and revealed that there was decrease in total feed intake (141.8 ± 5.31%) and total weight gain (9.11 ± 1.35%) in rats fed with basal diet and given acetaminophen when compared with those rats fed with basal diet without acetaminophen (total feed intake, 443.8 ± 9.40%; total weight gain, 14.5 ± 1.70%). However, there was a significant increase (*P* < 0.05) in total feed intake (44.2 ± 2.10–73.5 ± 3.80%) of rats fed with tofu curdled with various coagulants and given acetaminophen orally compared to those rats fed with basal diet and given acetaminophen. In addition, the total weight gain of rats fed with tofu curdled with various coagulants plus acetaminophen decreased significantly (−5.1 ± 1.01–2.1 ± 0.60%) when compared with those fed with acetaminophen (9.1 ± 1.35%).

The results of liver function tests of the rats studied are shown in Table 3 and revealed that serum AST, ALT, ALP, and LDH levels of rats treated with basal diet and given acetaminophen orally were quite higher than that of the group treated with basal diet without acetaminophen. In contrast, the rats treated with various tofus and acetaminophen orally had significantly lower levels of AST, ALT, ALP, and LDH when compared with the group treated with basal diet and acetaminophen orally (Table 3). 

 The results of serum chemistry of rats studied are presented in Table 4 and revealed that bilirubin (0.153 ± 0.12 mg/dL), cholesterol (243.45 ± 7.00 mg/dL), and glucose (162.68 ± 5.70 mg/dL) levels significantly (*P* < 0.05) increased in rats treated with basal diet without acetaminophen intubation when compared with those treated with basal diet and acetaminophen intubation. Nevertheless, the content of total protein (3.45 ± 0.80 mg/dL) and albumin (1.65 ± 0.57 mg/dL), respectively, significantly decreased (*P* < 0.05). In addition, rats fed with various coagulated tofu and acetaminophen showed significant (*P* < 0.05) reduction in the level of bilirubin, cholesterol, and glucose when compared with rats fed with basal diet and acetaminophen intubation. In contrast, the level of total protein and albumin of rats fed with various tofus and acetaminophen were significantly increased (*P* < 0.05) when compared with those fed with basal diet and acetaminophen.

## 4. Discussions

The antioxidant properties of polyphenolic extractions of tofu produced using different coagulants and the effects of these tofus on acetaminophen-induced hepatotoxicity in albino rats were evaluated. Polyphenols, particularly the flavonoids, are among the most potent plant antioxidants. Polyphenol can form complexes with reactive metals such as iron, zinc, and copper reducing their absorption [21]. It seems to reduce nutrients absorption, but excess levels of such elements (metals cations) in the body can promote the generation of free radicals and contribute to the oxidative damage of cell membranes and cellular DNA [25]. In addition, polyphenols also function as potent free radical scavengers within the body, where they can neutralize free radicals before they can cause cellular damage [26]. The results of polyphenolic antioxidant properties of various tofus studied as indicated in Table 1, revealed that steep water coagulated tofu had a significantly higher (*P* < 0.05) total phenol, followed by alum coagulated tofu, and calcium salt tofu had the least value of total phenol. These values generally compared well with what Shokunbi et al. [9] reported for some varieties of commercial mushrooms (0.01 g/100 g). The results are also in line with the report of Oboh and Rocha [27] on some hot pepper (*Capsicum annum, *Tepin, and *Capsicum pubescens*). In contrast, free radical scavenging activity of the free phenols of various types of tofu analyzed had a significantly higher (*P* < 0.05) calcium coagulated tofu, followed by alum coagulated tofu, and steep water coagulated tofu, which is the least (Table 1). However, the reverse was the case for the bound phenols where steep water coagulated tofu was recorded high, followed by alum coagulated tofu, and calcium-coagulated tofu had the least value of bound phenol (Table 1).

These values are higher than what Oboh and Akindahunsi [28] reported on some commonly consumed green leafy vegetables in Nigeria. Oxidative stress is the state of imbalance between the level of antioxidant defense system and the production of the oxygen-derived species. The increased level of oxygen and oxygen-derived species causes oxidative stress. The finding suggests that various tofus produced using different coagulants are good source of antioxidants and could be used to reduce biological oxidative stress and prevent cellular damage by scavenging free radical activity of the oxidative stress. Table 2 shows the results of total feed intake and total weight gain of rats studied. The result reveals that there was a decrease in total feed intact and total weight gain of the rats fed with basal diet and acetaminophen when compared with those rats fed with basal diet without acetaminophen (Table 2). This drastic reduction in feed intake and total weight gain could be because of overdose of paracetamol intake that generated oxidative stress in rats. The free radicals produced in rats might cause certain abnormality in food consumed and weight gain. It was also observed that rats fed with alum-coagulated tofu consumed the highest amount of tofu in the duration of the experiment, followed by those fed with calcium chloride coagulated tofu, while those fed with tofu coagulated with steep water consumed the lowest amount of tofu (Table 2). The total weight gain of rats fed with tofu produced using different coagulants was significantly higher (*P* < 0.05) in alum-coagulated tofu, and tofu coagulated with steep water significantly had the least weight gain (Table 2). The higher consumption of alum and calcium chloride coagulated tofu might be because of good taste and odour as indicated in the tofu, and the negative value of weight gain recorded by the rats fed with steep water tofu could be because of very low feed intake. While, the low intake of the tofu coagulated with steep water could be because of the unpleasant odor imparted by the steep water to the tofu. The wide variation for tofu consumed by the various rats could be because of the difference in the taste, nutritional quality, and acceptability of the various coagulated tofu [16, 29]. As shown in Table 3, overdosing rats with 100 mg/mL/day acetaminophen alone for 14 days caused a significant increase (*P* < 0.05) in the serum marker enzymes (AST, ALT, ALP, and LDH) (Table 3). Increase in AST levels signified liver damage. This finding suggested that the mega doses of acetaminophen administered induce the production of free radicals, which cause damage to the hepatocytes of rats. 

This result correlates with the finding of Eriksson et al., [30] that toxicity with acetaminophen occurs when too much of it is taken. The elevations of serum liver enzymes indicate liver damage. This correlates with the report of Sai et al., [31] that a significant increase in serum AST, ALT, and ALP levels suggests liver damage. Under normal therapeutic dose of acetaminophen, the excessive metabolites produced by the cytochrome P-450 system can be reduced by glutathione. However, if acetaminophen is overdosed, the glutathione stores will be depleted and the excessive metabolites will react with the liver macromolecules and cause hepatic cell death. The hepatic cellular enzyme ALP in serum will therefore increase. In addition, the hepatic malondialdehyde level will increase invariably, hence resulting in the generation of free radicals in the body [21]. This suggests that acetaminophen hepatotoxicity appears to be critically dependent on the depletion of cellular glutathione, and a relatively high reduction in the intracellular level of reduced glutathione leads to a situation of oxidative stress [32, 33]. Conversely, a simultaneous administration of albino rats with 100 mg/mL/acetaminophen alongside various tofus produced caused a significant decrease (*P* ≤ 0.05) in serum AST, ALT, and ALP (Table 3). Decrease in serum marker enzyme levels (liver enzymes) indicates the ability of various tofus to protect the hepatocytes from oxidative damage caused by overdosing rats with acetaminophen. This is an indication that antioxidant mechanism may be involved in the protection of the liver cell by the various tofus from acetaminophen-induced oxidative stress. It was suggested that soy products contain high antioxidant properties, which serve as an extracellular neutralizer of free radicals [34]. 

In addition, soy food and vegetables had been reported to be rich in many phenols such as flavonoid [35]. Flavonoids have antioxidants capacity that is much stronger than those of vitamins C and E, reportedly used to prevent free radical production [35]. In contrast, the serum LDH level of rats fed with basal diet and acetaminophen was increased significantly (*P* < 0.05) when compared with those fed with basal diet without acetaminophen (Table 3). This is in line with the findings of Ravikumar et al., [36] that the serum LDH level was elevated in rat administered CCl_4_. The increased level of LDH is an indication of abnormality in liver functioning, which may be due to the formation of highly reactive free radicals caused by acetaminophen overdose. The hepatotoxicity of acetaminophen may directly affect the polyunsaturated fatty acids and alter the liver microsomal membranes in the rats. In a reverse case, the LDH levels of rats fed with tofu and acetaminophen were significantly decreased (*P* < 0.05) when compared with that of rats in group BDA (Table 3), which is a sign of an improvement by the various tofus over the damage done to the liver by acetaminophen. The result of serum total cholesterol level as indicated in Table 4 revealed that there was a significant increase in total cholesterol of rats fed with basal diet and acetaminophen orally when compared to those fed with basal diet without acetaminophen orally administered (Table 4). 

The elevation in serum total cholesterol level could be attributed to the ability of acetaminophen to induce the production of free radicals, which results in hypercholesterolemia and the atherosclerosis. This correlates with the findings of Oboh [15] that an increase in the serum levels of cholesterol and LDL is associated with hypercholesterolemia and atherosclerosis, respectively. It is also supported by the findings of Ravikumar et al., [36] that intoxication of rat with CCl_4_ elevated total cholesterol levels, which suggests the inhibition of bile acid synthesis and leads to increased level of cholesterol. However, supplementation of tofu curdled with various coagulants and acetaminophen orally administered causes a significant decrease (*P* > 0.05) in serum total cholesterol, compared to those fed with basal diet with acetaminophen orally administered (Table 4). It indicated that tofus produced using three coagulated agents are capable of preventing acetaminophen inducing oxidative stress and inhibiting bile acids synthesis.

 This could be attributed to high antioxidant potential of soybeans, which serve as an extracellular neutralizer of free radicals [37]. This finding is in line with the study of Oboh [15] that the antihypercholesterolemic effect of soy protein was found to decrease the plasma concentrations of LDL as well as the ratio of plasma LDL to high density lipoprotein (HDL). It was reported that flavonoids could protect membrane lipids from oxidation, and a major source of flavonoids is vegetables, fruits, and soybeans [35]. The significant (*P* < 0.05) reduction in total protein and albumin levels in rats fed with basal diet with acetaminophen intubation, compared to those fed with basal diet without acetaminophen orally administered (Table 4), indicates cellular damage produced. The damage produced might be due to the functional failure of endoplasmic reticulum, which leads to decrease in protein synthesis and accumulation of triglycerides [36]. Increased serum bilirubin level in rats fed with basal diet with acetaminophen intubation (Table 4) could be looked upon as a compensatory/retaliatory phenomenon in response to cellular peroxidative changes, which cause damage to the biliary gland. This is because bilirubin functions *in vivo* as a powerful antioxidant, antimutagen, and an endogenous tissue protector [37]. Reduction of bilirubin and elevation of total protein and albumin levels in rats treated with various coagulated tofus and acetaminophen orally administered (Table 4) were most effective and stabilized the biliary cell function and endoplasmic reticulum leading to bile acid and protein synthesis [38]. This indicates hepatoprotection. The administration of acetaminophen alone may adversely interfere with protein metabolism probably by inhibiting the synthesis of proteins such as albumin in the liver. 

Simultaneous administration of acetaminophen to rats with supplementation of various tofus produced reversed these changes, maybe by increasing protein synthesis. Stimulation has been advanced as a contributory hepatoprotective mechanism, which accelerates regeneration of cells [39]. LDH activities all point to the fact that tofu has hepatoprotective potential against hepatotoxin caused by acetaminophen. It could be possible that a probable mechanism of hepatoprotection of various tofus against acetaminophen-induced damage is the antioxidant activity. The antioxidant activity of the various tofus supplemented may be attributed to the presence of phenolics and flavonoids [40]. Therefore, lower level of serum enzyme markers, total cholesterol, and bilirubin observed in rats on tofu coagulated with various coagulants and acetaminophen orally administered suggested liver damage repair, but steep water coagulated tofu looked more promising in terms of liver repair than other forms of tofu diet. It can be concluded from the present finding that tofus curdled with various coagulants could be efficiently used to prevent liver damage caused by high doses of acetaminophen orally administered after successful clinical trials. In addition, the intake of acetaminophen in excess should be discouraged in homes because of its destructive effects on the liver. Tofu consumption should be encouraged in diets as it can be used as a functional food to prevent liver damage due to its antioxidant properties, and as such, tofu could be recommended for clinical trial.

## Figures and Tables

**Table 1:** Antioxidant properties of tofu studied.

Sample (tofu)	TP	FSFP (%)	FSBP	RPFP	RPBP
SWT	15.0 ± 2.24^a^	38.2 ± 3.57^c^	69.1 ± 4.80^a^	0.4 ± 0.20^a^	0.4 ± 0.20^b^
ALT	13.0 ± 2.08^b^	47.3 ± 3.97^b^	56.5 ± 3.36^b^	0.3 ± 0.17^b^	0.5 ± 0.30^a^
CST	11.0 ± 1.91^c^	65.5 ± 4.67^a^	27.3 ± 3.02^c^	0.4 ± 0.20^a^	0.4 ± 0.20^b^

Values with the same superscript letter(s) along the same column are not significant different (*P* ≤ 0.05). SWT: steep water coagulated tofu, ALT: alum coagulated tofu; CST: calcium salt coagulated tofu' TP: total phenol; FSFP: free radical scavenging ability of free soluble phenols; FSBP: free radical scavenging ability of bound phenols; RPFP: reducing power of free soluble phenols; RPBP: reducing power of bound phenols.

**Table 2:** Total feed intake and weight gain of albino rats fed with commercial diet and various coagulated tofu (g/rats/days).

Sample	Total food taken (%)	Total weight gained (%)
BDA	141.8 ± 5.31^b^	9.11 ± 1.35^b^
BDWA	443.8 ± 9.40^a^	14.5 ± 1.70^a^
CSTA	64.7 ± 3.60^d^	−3.2 ± 0.80^d^
SWTA	44.2 ± 2.10^e^	−2.1 ± 0.60^e^
ALTA	73.5 ± 3.80^c^	−5.1 ± 1.01^c^

Values represent mean of triplicate reading. Means with the same superscript letter(s) within the same column are not significantly different (*P* ≤ 0.05). BDA: basal diet with acetaminophen orally administered; BDWA: basal diet without acetaminophen orally administered; ALTA: alum coagulated tofu with acetaminophen; SWTA: tofu coagulated with effluent from pap with acetaminophen; CSTA: calcium coagulated tofu with acetaminophen orally administered.

**Table 3:** Serum enzyme marker level of rats studied.

Sample (rats)	ALP (mg/dL)	AST (mg/dL)	ALT (mg/dL)	LDH (mg/dL)
BDA	122.0 ± 4.93^a^	101.0 ± 4.49^a^	31.0 ± 2.49^a^	156.00 ± 5.59^a^
BDWA	90.0 ± 4.24^c^	77.0 ± 3.92^c^	26.0 ± 2.28^b^	78.40 ± 3.96^c^
SWTA	110.0 ± 4.70^b^	82.0 ± 4.05^b^	26.0 ± 2.28^b^	85.52 ± 4.14^c^
ALTA	120.0 ± 4.90^ab^	94.0 ± 4.34^b^	27.0 ± 2.32^b^	90.00 ± 4.24^b^
CSTA	113.0 ± 4.75^b^	80.0 ± 4.0^c^	30.0 ± 2.45^ab^	90.23 ± 4.25^b^

Means with the same superscript letter(s) along the same column are not significantly different (*P* ≤ 0.05). BDA: basal diet with acetaminophen orally administered; BDWA: basal diet without acetaminophen orally administered; SWTA: steep water coagulated tofu with acetaminophen orally administered; ALTA: alum coagulated tofu with acetaminophen orally administered; CSTA: calcium chloride coagulated tofu.

**Table 4:** Serum chemistry level of rats studied.

Sample (rats)	BIL	CHL (Mg/dL)	GLU	TPN	ALB
BDA	0.153 ± 0.12^a^	243.45 ± 7.00^a^	162.68 ± 5.70^a^	3.45 ± 0.83^d^	1.65 ± 0.57^d^
BDWA	0.125 ± 0.14^b^	95.20 ± 4.36^d^	87.34 ± 4.18^c^	7.75 ± 1.24^a^	3.93 ± 0.89^a^
SWTA	0.118 ± 0.04^c^	145.32 ± 5.40^c^	93.50 ± 4.32^b^	7.05 ± 1.19^b^	3.00 ± 0.77^b^
ALTA	0.122 ± 0.12^c^	157.50 ± 5.61^b^	95.00 ± 4.36^b^	6.45 ± 1.14^c^	2.60 ± 0.72^c^
CSTA	0.128 ± 0.16^b^	148.43 ± 5.45^c^	93.76 ± 4.33^b^	6.74 ± 1.16^c^	2.91 ± 0.76^c^

Means with the same superscript letter(s) along the same column are not significantly different (*P* ≤ 0.05). BDA: basal diet with acetaminophen orally administered; BDWA: basal diet without acetaminophen orally administered; SWTA: steep water coagulated tofu with acetaminophen orally administered; ALTA: alum coagulated tofu with acetaminophen orally administered; CSTA: calcium chloride coagulated tofu; BIL: bilirubin; CHL: cholesterol; GLU: glucose; TPN: total protein; ALB: albumin.
